# An Improvement in the Identification of the Centres of Checkerboard Targets in Point Clouds Using Terrestrial Laser Scanning

**DOI:** 10.3390/s19040938

**Published:** 2019-02-22

**Authors:** Anna Fryskowska

**Affiliations:** Department of Remote Sensing, Photogrammetry and Imagery Intelligence, Geodesy Institute, Faculty of Civil Engineering and Geodesy, Military University of Technology, 00-908 Warsaw, Poland; anna.fryskowska@wat.edu.pl; Tel.: +48-623-839-692

**Keywords:** point cloud registration, target identification, laser scanning, geomatics, remote sensing

## Abstract

Measurement using terrestrial laser scanning is performed at several stations to measure an entire object. In order to obtain a complete and uniform point cloud, it is necessary to register each and every scan in one local or global coordinate system. One registration method is based on reference points—in this case, checkerboard targets. The aim of this research was to analyse the accuracy of checkerboard target identification and propose an algorithm to improve the accuracy of target centre identification, particularly for low-resolution and low-quality point clouds. The proposed solution is based on the geometric determination of the target centre. This work presents an outline of a new approach, designed by the author, to discuss the influence of the point cloud parameters on the process of checkerboard centre identification and to propose an improvement in target centre identification. The validation of the proposed solutions reveals that the difference between the typical automatic target identification and the proposed method amounts to a maximum of 6 mm for scans of different qualities. The proposed method may serve as an alternative to, or supplement for, checkerboard identification, particularly when the quality of these scans is not sufficient for automatic algorithms.

## 1. Introduction

For topographic applications, Terrestrial Laser Scanning (TLS) uses medium-range equipment. Measurements are performed at several stations to measure an entire object. Each scan is conducted in the local scanner coordinate system, which is directly related to the position of the instrument. In order to obtain a complete and uniform point cloud, it is necessary to orient each and every scan to one common local or global coordinate system. In the case of laser scanning data, this process is referred to as the scan registration. One of the methods for the scan registration of independently obtained data is the transformation of the systems on the basis of reference points—in this case, the points that are common in both data sets. These can be both natural points, such as roof corners, edges, and other elements of topographic objects, or signalized points in the form of LiDAR targets or ground markers [1,2,3,4,5]. Signalized points are usually pre-defined targets/marks with known geometry, shape and pattern. The points can be used for automatic or manual registration. In publication [6], the efficiency of different registration methods was validated.

**Figure 1:**
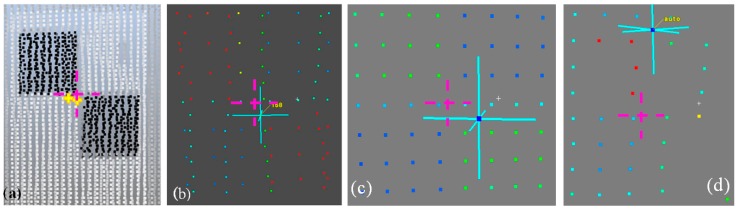
Identification of the target centre (**a**) automatically (yellow crosses) on two different dense point clouds; (**b**) the manual identification of the centre in a sparse point cloud; (**c**,**d**) erroneous centre autodetection. Fuchsia crosses indicate theoretical target centre.

In the case of TLS, there are a few typical artificial reference objects. The selected targets and their representation on the point cloud are presented in Figure 1.

**Figure 2:**
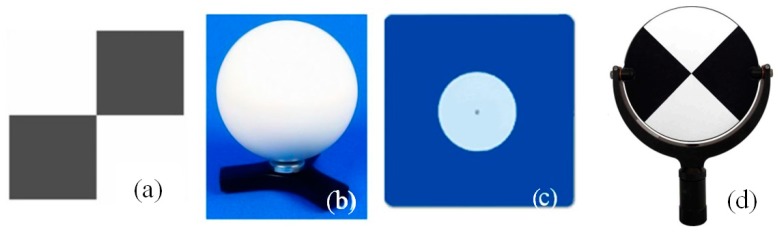
Typical targets: (**a**) checkerboard, (**b**) sphere, and (**c**) HDS target [5].

The principal advantage of using reference spheres is the possibility of scanning them from various angles. They also guarantee a uniform reference surface. The sphere’s mass centre is determined on the basis of a point cloud that represents its surface. The main disadvantage is that they are difficult to use in georeferencing, namely, returning them to the centre of the target and its main surface. Spherical targets are omnidirectional; nevertheless, the accuracy of extracting sphere parameters is range-dependent [7]. In turn, checkerboard targets became the most common and universal among all the available artificial reference targets. Checkerboards are usually printed targets, with squares arranged in two alternating colours (white and black). The accuracy of the centre identification of the scanned checkerboards may be higher than that of spheres (considering the same point resolution and conditions); however, the measurement process itself is more problematic. Checkerboard targets are immobile and require the right scanning sampling. High Definition System (HDS) targets (Figure 2c) are objects that are specifically designed for the material from which they are made. These materials have different reflectance coefficients. On the other hand, checkerboard printed targets are cheaper and more universal, for example, when combing different data types of scans and images.

With almost all kinds of objects, the centres of the reference points are extracted from the scan point clouds. Due to the irregular distribution of the points in a point cloud, the selection and determination of characteristic elements is ambiguous. Spare point distribution is always different in each separate scan. Therefore, it can be concluded that it is impossible to define the exact same points for two or more scans. First, the points are never identical, so they are referred to as pseudo-homologous points; secondly, this ambiguity results mainly from the nature of laser data and the varying resolution of both data sets.

Figure 1a shows an example of the automatic identification of the target centre from two different scans, with an algorithm that is applied in dedicated software. In the results, we obtain two different centre positions. Similarly, inaccurate manual centre identification might also be erroneous in sparse point clouds (marked with blue crosses in Figure 1b–d; fuchsia crosses indicate the theoretical target centre).

For the efficient registration of scans of different qualities, it is desirable to estimate the above-mentioned target centres automatically, with a high precision. Many automatic methods have been developed by both scanners and software vendors for each processing type. Automatic detection algorithms are mostly based on the intensity values and identification of, e.g., edges or normals of the target edges. While they work correctly, it is evident that their accuracy depends on the resolution and quality of the data. Nevertheless, the primary drawback of these algorithms is that the processing results depend strongly on the quality and resolution of the point clouds. Of course, there are some algorithms to improve homologous point identification, for example, the Iterative Closest Point (ICP) method is mostly dedicated to the cloud-to-cloud registration methods [6,8]. Without good initial values, these methods do not work well or efficiently [9]. The authors of the first publication regarding this method were Besl and McKay [10], who, in 1992, based their method on the Horn method. At the same time, in that same year, further works appeared on the same method [11,12,13,14]. The latest document regarding the changes and modifications of the ICP algorithm, during the years 2002–2007, is the work of Wild [15]. Since then, the algorithm has undergone many modifications [16]. The ICP method is simple and fast, which allows it to be easily used in the elaboration of data. The disadvantage of the method is its sensitivity to gross errors and interferences in the data. Of course, the adverse impact of gross errors can be compensated by data filtration, performed before the main data processing. Another downside of the method is its inability to match points in point clouds with different densities (resolutions), noise, and unfiltered and low coverage [9]. Therefore, many improvements of this algorithms have been introduced. For example, in paper [17], the The Picky ICP algorithm was developed, which has been created by merging several extensions of the standard ICP algorithm, thus improving its robustness and computation time. Similarly, Bae and Lichti [18] proposed a method for the registration of two point clouds, the Geometric Primitive ICP with the RANSAC (GP-ICPR), and new, advanced improvements of this method can be found in [19,20,21]. Nevertheless, ICP and similar algorithms are very popular in cloud-to-cloud scan registration and were not used in this paper.

### 1.1. Related Works

There is not much research into the literature describing the problem of identifying targets, although automatic recognition and measurement of targets is implemented in a lot of data processing software. The instruments are capable of preliminarily localizing the rough target position, and consequently, high-resolution scanning is performed in the vicinity. When a target is scanned, the acquisition software automatically calculates the centre of it by applying an algorithm for its measurement. Nevertheless, the algorithms implemented in commercial software are unknown for the most part [22]. Various methods relating to the identification of the centre of the target, on the basis of, e.g., information about the radiometric centre of all returns or point of maximum radiance, are presented in the literature [23]. The coordinates of the centre should be estimated from a number of laser returns covering the surface of the target. This estimation process will introduce target reduction error [24]. What is more, when detecting targets—particularly those using the intensity parameter—some discrepancies related to beam reflection, such as the halo effect, can occur. The halo effect presents the problem of how to estimate the position of the target centre from a cluster of responses. This intensity-based weighting strategy implicitly assumes that the strongest signal should be returned from the centre of the reflective target [25]. Some other approaches using the fuzzy clustering technique, introduced by Bezdek in [26] and developed by Valanis and Tsakiri in [27], may be found. Intensity investigations were conducted in [28] that present a general approach to intensity normalization, considering the diffuse and specular scattering characteristics of the surface. Such an assumption could be proved by investigating reference targets, where the backscattering characteristic is known or could be measured by reference measurements. Meanwhile, Wang and Brenner applied the SIFT method in the extraction of feature points from the reflectance image and geometric constraint to exclude false matches [29]. Nevertheless, all of these methods involve the intensity value as the most influential parameter.

Target identification is one of the basic tasks in the process of scan registration or georeferencing. For example, in [30,31], the problem of the accuracy of reference points used in georeferencing is described. Therefore, this article will discuss the question of the accuracy of identifying the centre of checkerboard targets for targets with a low scanning quality. Of course, target identification is not only a problem during registration, but it also plays a very important role in point distribution. The registration effect of target distribution is the main topic of publication [32].

#### 1.1.1. Target Identification Problem and Research Background

Manufacturers and distributors of software for obtaining and processing data from terrestrial laser scanning provide recommendations for obtaining such targets. One of them is that the laser’s angle of incidence should not be more than 45°. Depending on the chosen scanning resolution, the automatic detection of checkerboard references is unreliable beyond a certain distance from the scanner. For example, when using A4 checkerboard references and scanning with a resolution of 1/4, the distance to the scanner should not be greater than 15 m [33]. Moreover, to detect the checkerboard, the software (i.e., Faro Scene) needs at least four scan points on each quadrant (black/white area). The centre of the checkerboard target is calculated by means of edge detection. The better the line between black and white is identified, the greater chance that the calculated centre will be in the real centre. The same distributor has determined scan parameters and the distance to the station for optimal measurement quality. Two black and white checkerboard targets in two versions of the target size were compared. The objective was to determine how the detection of targets behaves with increasing distance from the scanner. That means that we simply click on the target, and an algorithm searches for the intersection point of both black rectangles [34].

The detection of small targets by means of automatic target detection works very well at a distance of 7.5 m. Detection works for almost all of the selected resolutions. It is only at 1/32 resolution that the 7.5 m distance provides too few points to determine the target’s centre. As might be expected, the visibility decreases with increasing distance. At a distance of 35 m, the small target can easily be detected at 1/2 resolution [35].

**Figure 3:**
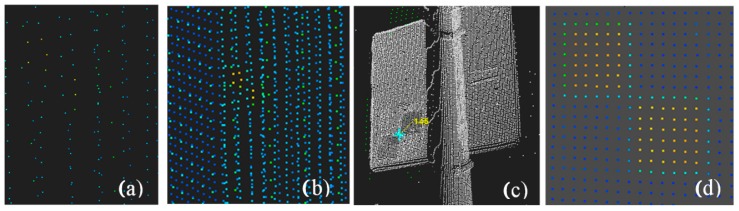
Target scans with different representation qualities. (**a**,**b**)–sparse points; (**c**,**d**)–dense point clouds.

Nevertheless, in practice it is difficult to preserve the above measurement conditions; thus, this article will examine the possibility and accuracy of identifying checkerboard targets and their impact on the registration quality. Point clouds representing checkerboard targets with different scan qualities are presented in Figure 3.

The quality consists of: the point cloud resolution, point distribution pattern (regularity) and completeness (missing structures). These parameters will later determine the choice of processing method.

Of course, the software dedicated to the processing of point clouds has its own embedded search algorithms for such points and targets, but they are not explicit. In addition, targets are not always identified or are embedded incorrectly in the selected scan point. Nevertheless, the basic factors affecting the quality of target detection are the spatial resolution and the interaction of the laser beam with the object.

#### 1.1.2. Resolution

One of the most important parameters for the detection of objects in a point cloud is the cloud’s resolution, understood as the distance between the neighbouring points. The distance depends on the type of scanning system, its technical data, and measurement parameters, including surface sampling. In the case of terrestrial laser scanners, it is possible to achieve an average distance between the points at a level of 1–2 mm; however, in practice, the values of 0.5–3 cm (static scanners) and 1–8 cm (mobile scanners) are used [36,37,38,39,40].

#### 1.1.3. Laser Beam—Object Interaction

In the case of TLS data, a single return is registered. Even in favourable data-obtaining conditions, the laser footprint during the reflection of a light beam from the object surface will have a certain size, and depending on the type of surface and scanning angle, artefacts, such as noise or mixed pixels, may occur, generally indicating partial or multiple returns from nearby objects [41]. The value of the return intensity is also related to the material from which the surface of the object is made. The scanning angle and distance will then influence the energy/intensity of the return. Research related to the determination of the impact of the abovementioned factors on the quality and intensity of the returning beam has been published in several papers [28,42,43,44,45,46].

### 1.2. Research Purpose

This article will discuss the question of the accuracy improvement of identifying the centre of checkerboard targets for targets with a low quality (low resolution, irregular point distribution), particularly for scans where recommended scanning parameters could not have been applied. The correct determination of reference points is of particular importance in the processing of georeferencing scans. Therefore, the results were referenced to the data and measured using a total station instrument.

The aim of this research was to analyse the accuracy of checkerboard target identification and propose an algorithm to improve the accuracy of target centre identification, particularly for low-resolution and low-quality point clouds, when automatic algorithms cannot detect the target centre. The proposed solution is based on the geometric determination of the target centre but takes into account different point cloud quality parameters. Point intensity (radiometry) serves as a support rather than a basis for identification.

The article discusses the influence of the point cloud parameters on the process of checkerboard centre identification, presenting the results of the proposed method and an accuracy analysis based on reference data.

## 2. Materials

The analysed point clouds were obtained on a 650-metre-long street in the old district of the city. The studies were conducted by means of a phase-based terrestrial laser scanner (Focus 3D, Faro, Lake Mary, FL, USA). Reference data used for accuracy assessment were measured using a GPT-3100N5 reflectorless total station (Topcon, Tokyo, Japan) and a Sprinter 250M bar code level (Leica Geosystems AG, Heerbrugg, Switzerland). A topographic network was established, to which the check measurements referred.

**Figure 4:**
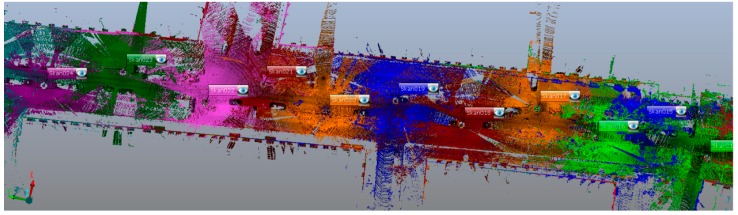
Fragment of obtained street data (stations marked by different colours).

The checkerboard targets were selected for the analyses and arranged on different elements of the test area: building walls, road signs, poles, lampposts, etc. They were to be used for orienting every station for scanning, conducted on section of a narrow street that was about 650 m long. Due to the geometry of the station sequence and the distribution of objects to be scanned (buildings and elements of road infrastructure), the targets could not always be situated in the recommended manner (angle and distance—Figure 4). In narrow street point distribution, possibilities are limited, though this aspect is very important [32]. Thus, the analysis concerned targets both favourably and unfavourably positioned in relation to the measuring instrument as well as the type and flatness of the ground.

The scan was conducted with a resolution of ca. 10 mm at 20 m. Over 80 checkerboard targets were scanned, together with all objects. The coordinates of the check points were determined in the local and global national coordinate system (PL-2000, and the heights in the PL-EVRF-2007-NH system). Points were to be reference points for point cloud registration in the local coordinates system and georeferencing in the global one. The accuracy of the reference (and check) point determination of the global coordinate system is between 5 mm (2D) and 10 mm (3D), which is related to the topographic network. For further analysis, the topographic network points are considered as reference measurements.

### Types of Target Scan Quality

**Figure 5:**
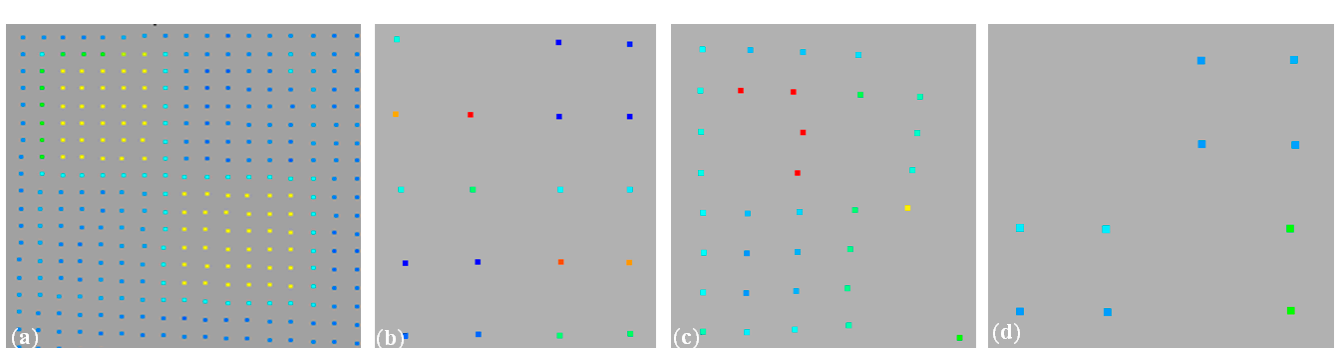
Scans of different qualities: (**a**) 142 regular point distribution targets (**b**) 99 regular scans of a lower density, (**c**,**d**) 97 irregular scan points, and the low completeness level is 106.

Data from measurement sets were used for the analysis. Different target scan qualities are considered:

(1) Regular, high-resolution target scan: Complete, dense point cloud representing a checkerboard (Figure 5a);
(2) Regular scan points of a lower quality: Sparse point cloud but complete representation of the target (Figure 5b)—such quality still causes problems in target identification;
(3) Irregular scan points, and low completeness level: Incomplete, low scan quality (Figure 5c,d).

Usually for targets of a very low resolution or number of points in each target field, it is not possible to detect them automatically. In the case of the target in Figure 5d, both the resolution and cloud quality are relatively low, which hinders both automatic and manual centre detection. The target was obtained from an unfavourable and not recommended angle (less than 45^o^), from a distance of more than 20 m.

## 3. Methods and Description of the Approach

Below, the proposed workflow has been introduced. At the same time, for each processing step, some results are presented on selected examples.

### 3.1. Research Framework and Overview of the Proposed Method

**Figure 6:**
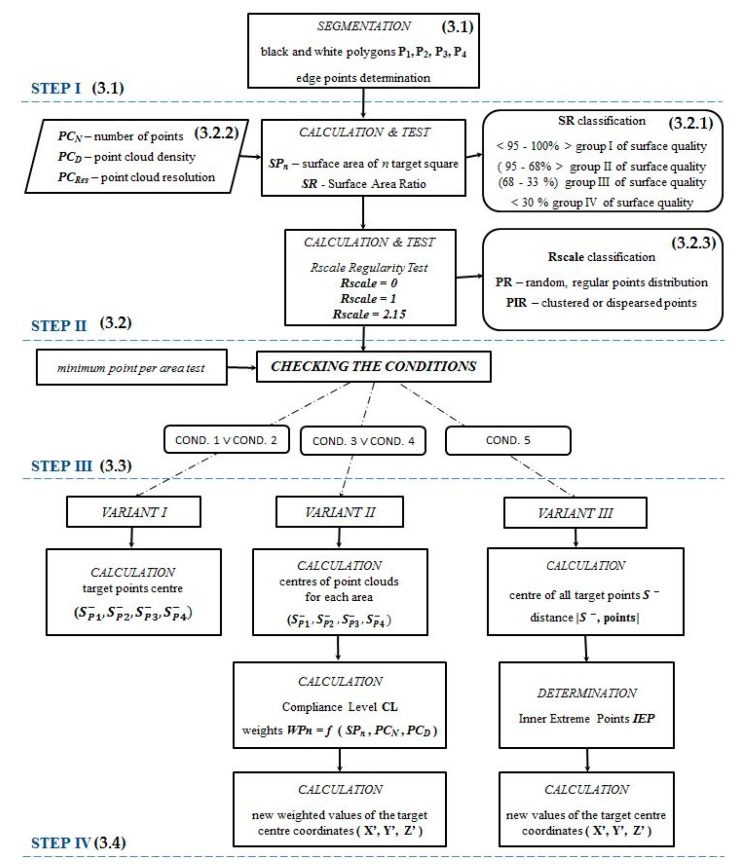
Overview of the methods for the developed approach. Numbers in brackets represent the corresponding sections in this paper.

The proposed scheme used the concept of point-based target identification, employing the weighting of point cloud quality parameters. Figure 6 shows the workflow of the proposed scheme.

In the method, four main processing steps can be distinguished:

*Step I* is a preliminary processing step, which includes preparing the input data, meaning point clouds representing the checkerboard target area. During the segmentation process (described in Section 3.1), black and white polygons and edge points are determined.

*Step II* is the calculation of the proposed point cloud parameters and developed coefficients. These are: point cloud density (PC_D_), resolution (PC_Res_), surface area ratio (SR) and a regularity test, leading to point cloud classification in a distribution condition (PR and PIR). These parameters are direct indicators for point cloud quality and are based on decisions on which variant of the target centre calculations to choose. A description of the proposed quality parameters and the point cloud quality classification are introduced in Section 3.2.

The decision process is included in *Step III* and precisely described in Section 3.3.

*Step IV* is a final step, resulting in calculations of target centres according to variants chosen in the previous stage. Variants are precisely described in Section 3.4.

All the variants are based on the analysis of all points identified as target fields, but with due regard to the points with a higher or lower quality. The fundamental element of the method is the weight assigned to every point group, based on which the target centre is determined. Weights are chosen considering the above-mentioned point cloud quality parameters.

In this paper, a comparison of the well-known manual and automatic method of target identification, with proposed algorithms, is conducted. Target centres can be identified using one of the 5 following methods. The first is manual operator interpretabilities and skills and the second is automatic method – implemented in the software dedicated to particular scanning system. Methods 3-5 are proposed methods:

(1) Manual (MAN)—point representing the target centre chosen by the operator. Selection of one of the points representing the target.
(2) Automatic (AUTO)—determining the target centre using an algorithm based on field edge detection. As in the previous method, the target centre is also one of the existing cloud points.
(3) Weighted mean values (WMV)—method proposed by an author for determining the target centre by means of selected weighting scan parameters (variant II). Using this method, the data are processed in order to determine the point where the target centre should be located. It is not one of the points existing in the set but is calculated on the basis of other points representing the object.
(4) Weighted mean of the inner extreme points (WIP)—proposed method for determining the target centre by means of weighting scan parameters and/or “extreme inner” points (variant III in the diagram in Figure 6). In this approach, similarly to the WMV method, the centre point is calculated from the data set.
(5) Terrain reference measurements (TR)—checking the total station measurements representing the ground truth. The measured points are real points of the intersections of the black and white target fields (squares).

The consecutive paper sections contain precise descriptions of the processing steps presented in Figure 6.

### 3.2. STEP I—Preliminary Processing of Input Data

Before considering the particular variants, specific preliminary processing steps, common to all of the variants, are conducted in the first stage.

#### 3.2.1. Checkerboard Segmentation

**Figure 7:**
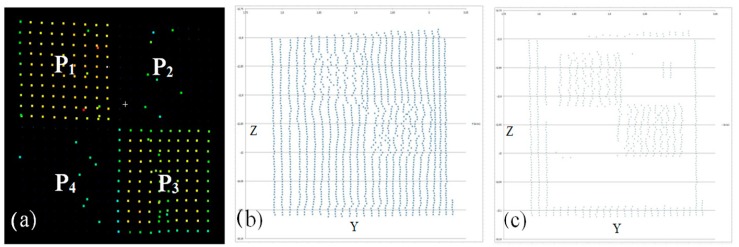
Point cloud segmentation based on the intensity parameter: (**a**) point clouds after intensity thresholding; (**b**) all target points reduced to 2D; and (**c**) points after intensity segmentation with noise.

In the first step, points belonging to and representing the black and white checkerboard fields are detected. It can be done semi-automatically or using other automatic target area sub-selection. The segmentation is based on intensity thresholding. Threshold value should be chosen respectively to data statistic. The appropriate threshold was determined from each test sample as a mean value, but sometimes it did not classify all points correctly (Figure 7b,c). Some erroneous observations of the targets had to be excluded by conducting the filtering step again for the entire dataset. Some of the erroneous measurements were also filtered using gross error limitation processing. Nevertheless, removal using the intensity value is useful when the point clouds are attached to the background of the same reflectance characteristics (for example, a white or black wall) (Figure 7a).

Then, the points are filtered for noises and artefacts on both the YZ and, separately, X planes. Any algorithms can be used to remove the noise. In this case, the Region Grow algorithm was used, which takes into account such parameters as the region thickness, maximum gap to span, and region size.

In the next step, the point cloud is reduced to 2D in order to simplify the calculations. After filtering and denoising, the third dimension, “depth,” of the target scan can be disregarded, assuming the flatness and low roughness of the target surface. On the basis of the intensity of the reflection and the arrangement of the four checkerboard fields, four separate fields (two white and two black) are determined. Then, the edge (boundary) points of each field are determined. For further analysis, separate fields are labelled P1, P2, P3 and P4, numbered counter-clockwise (Figure 7a).

Of course, in this step, other aggregation algorithms could be applied, for example, RANSAC, which is capable of extracting a variety of different types of primitive shapes, while remaining robust, general and simple. This algorithm was successfully applied in engineering tasks dealing with point clouds. The results can be seen in [41,47,48].

#### 3.2.2. Edge Points Determination

The boundary points of individual fields after segmentation will be successive vertices of polyhedrons or polygons. Then, we can consider the point cloud as a partially ordered set. However, at the stage of data processing in the form of sets of points, we can also specify some of them as the minimum and maximum elements in a partially ordered set. These minimum and maximum elements can be vertices of black and white squares.

**Definition** **1.** *Partially ordered set*

*A partially ordered set is defined as an ordered pair (D,≼), s is called the ground set, and ≼ is the partial order. p_i_—stands for the elements of the set.*

An element of a partially ordered set is the minimal element, when there are no elements in the set that are smaller than this element.

**Definition** **2.** *Convex set*

*If D is a linear space over the field of real numbers, set W⊆D is a convex set, if $\forall p_{x},p_{y}\in D\forall \left(\alpha_{1},\alpha_{2}\geq 0\;\wedge \alpha_{1}+\alpha_{2}=1\right)\left(\alpha_{1}p_{x}+\alpha_{2}p_{y}\right)\in W$.*

Empirically, this definition means that, for every pair of points within the set, every point on the line segment that joins the pair of points is also within the set.

Thus, the extreme points of a convex set can be determined either manually, or by means of search algorithms for the maximal and minimal points in a partially ordered set. The output set is then a point cloud, and the partial order relationship is made up of the following relations:

(1) $$\begin{array}{c} ≼:\left(x_{1},y_{1}\right)≼\left(x_{2},y_{2}\right)⟺x_{1}<x_{2}\\ ≼:\left(x_{1},y_{1}\right)≼\left(x_{2},y_{2}\right)⟺x_{1}>x_{2}\\ ≼:\left(x_{1},y_{1}\right)≼\left(x_{2},y_{2}\right)⟺y_{1}<y_{2}\\ ≼:\left(x_{1},y_{1}\right)≼\left(x_{2},y_{2}\right)⟺y_{1}>y_{2} \end{array}$$

Points that fulfil the above relationship are the appropriate vertex points; however, they might be points that fulfil the above relations only partially, while globally belonging, e.g., to the vertex of the square. Such points should be excluded from further analysis [2].

An alternative existing method is the implementation of Andrew’s [49] monotone chain algorithm:

A chain $C=\left(u_{1},\;\ldots ,\;u_{p}\right)$ is a straight-lined graph, with a set of vertices $\left\{u_{1},\ldots ,u_{p}\right\}$ and a set of edges $\left\{\left(u_{i},u_{i+1}\right):i=1,\;\ldots ,\;p-1\right\}$. The chain $C=\left(u_{1},\;\ldots ,\;u_{p}\right)$ is considered to be monotonic relative to the *L* line. If the line is an orthogonal line, *L* intersects *C* at exactly one point.

**Figure 8:**
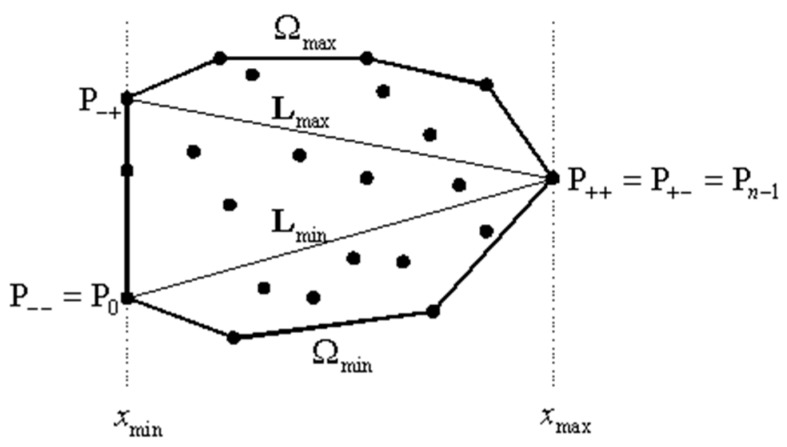
Convex hull for a point set and points defined: original point cloud - black dots, Ω - convex chain, lines *L*_min and *L*_max connect external, extreme points (*P*_minmin, *P*_minmax, *P*_maxmin, *P*_maxmax). [49].

First, the algorithm sorts the point set $S=\left\{P_{0},P_{1},\ldots ,P_{n-1}\right\}$ by increasing the value of x and y coordinates. Let the minimal and maximal x coordinates be marked as $x_{\min}$ and $x_{\max}$, respectively. Of course, $P_{0}.x=x_{\min}$, but there might exist other points with the minimal value of the *x* coordinate. Let $P_{\mathrm{minmin}}$ be a point of the *S* set, with *P*.*x* = *x*_min_ and then with the minimal *y* value among those points. Similarly, let $P_{\mathrm{minmax}}$ be a point, with $P.x=x_{\min}$ and then the maximal *y*. It should be noted that $P_{\mathrm{minmin}}=P_{\mathrm{minmax}}$ if there exists one unique point with a minimal *x* value. Points $P_{\mathrm{maxmin}}$ and $P_{\mathrm{maxmax}}$ are similarly defined as those which first assume the maximal *x* coordinate value, and then assume either the minimal or the maximal *y* coordinate value, respectively. Again, $P_{\mathrm{maxmin}}=P_{\mathrm{maxmax}}$ if there exists one unique point with the maximal *x* coordinate value. Then, points $P_{\mathrm{minmin}}$ and $P_{\mathrm{maxmin}}$ are connected in order to define line $L_{\min}$. Similarly, points $P_{\mathrm{minmax}}$ and $P_{\mathrm{maxmax}}$ are defined in order to define line $L_{\max}$. Those points and lines are shown in the diagram above (Figure 8). In the next step, the algorithm uses the vertices to create a convex chain $\Omega_{\min}$ under $L_{\min}$ connecting points $P_{\mathrm{minmin}}$ and $P_{\mathrm{maxmin}}$. Similarly, a chain $\Omega_{\max}$ over $L_{\max}$ is created, connecting points $P_{\mathrm{minmax}}$ and $P_{\mathrm{maxmax}}$. Then, the convex hull Ω of the set *S* is created by connecting $\Omega_{\min}$ and $\Omega_{\max}$ (Figure 8).

### 3.3. STEP II—Calculation the Basic Point Cloud Quality Parameters

#### 3.3.1. Surface Area and Surface Area Ratio 

Based on the separated sets of points representing the corresponding squares and their boundary (edge) points, the surface areas of the subsequent polygons are calculated. The polygon area can be determined using Gaussian formulas:

(2) $$\begin{array}{c} 2P=\sum X_{i}\cdot \left(Y_{i+1}-Y_{i-1}\right)\\ -2P=\sum Y_{i}\cdot \left(X_{i+1}-X_{i-1}\right)\\ SP_{n}=\frac{P}{2} \end{array}$$

where *SP_n_*—area of the square of interest, *P*—area of sub-polygons, *i* − 1—previous point index, *i* + 1—next point index, *i*—current point index, and *X, Y*—point coordinates. Of course, for the control, some conditions have to preserved:

(3) $$\begin{array}{c} \sum \left(Y_{i+1}-Y_{i-1}\right)=0\\ \sum \left(X_{i+1}-X_{i-1}\right)=0 \end{array}$$

Surface areas of the individual fields/squares (*SP*_1_, *SP*_2_, *SP*_3_, *SP*_4_) are then compared to the theoretical values (*TSP*_1_, *TSP*_2_, *TSP*_3_, *TSP*_4_). The theoretical area of every field of the target is known if we know the real dimensions of the target used. Consequently, the Surface Area Ratio (*SR*) is calculated as their quotient:

(4) $$SR=\frac{SP_{n}}{TSP_{n}}\cdot 100\%$$

I suggest the following classification of the SR values:

(a) 〈95÷100%〉—fields classified as group I of the surface quality (*SRC*_1_)
(b) (95÷68%>—fields classified as group II of the surface quality (*SRC*_2_)
(c) (68÷33%)—fields classified as group III of the surface quality (*SRC*_3_)
(d) <33%—fields classified as group IV of the surface quality (*SRC*_4_)

These surface area ratios will be used in calculating the weights in the final *Step IV*.

#### 3.3.2. Basic Quality Parameters of the Point Cloud—Number and Density of Points

Apart from the surface area, the other indirect quality metrics are the number of points and cloud density. Both parameters, and the Surface Area Ratio, provide indirect information about the regularity of the distributed points and the quality of the points representing the given checkerboard field. Therefore, the following symbols will be used later in this article: *PC_N_*—number of points in the cloud, *PC_D_*—point cloud density (number of points in the polygon area), *PC_Res_*—point cloud resolution (understood as an average distance between neighbouring points in the cloud), and *SP**_n_***—the surface area of *n* target square.

Presented in Table 1 are the example results of the parameters, calculated for the selected targets, scanned with different qualities.

#### 3.3.3. *Rscale* Regularity Test

Of course, as we can see in Table 1, an area bounded by extreme points, determined on the basis of the convex hull method, may not have the appropriate point number, density or point distribution. Point Pattern analysis seems to be obligatory because of the regularity or irregularity of the spatial distribution of points. Among many methods, in this case, it is reasonable to use the simplest and most popular of TLS applications [50], the method based on Nearest Neighbour Analysis. One of the developed methods is calculating the so-called Clark Evans statistics. The Clark Evans test is designed for areas with simple boundaries, preferably close to the square, because with the increase of irregularities of the area, the length of boundaries increases, which intensifies the boundary effect. Authors of this method showed that for a homogeneous Poisson distribution, for which the spatial process is isotropic, the average closest distance to the neighbouring object depends only on the density of events in a given area and is:

(5) $$r_{exp}=\frac{1}{2\sqrt{\frac{N}{TSP_{n}}}}$$

where *N* is the number of points in the distribution, and *TSP_n_* is the theoretical area of interest. Therefore, *r_exp_* is the expected average distance between the nearest neighbours, as determined by the theoretical pattern being tested.

In turn, according to [51], *r_obs_* is the observed average distance between the nearest neighbours. To measure the observed average distance between the nearest neighbours, we can calculate the distance between each point and all of its neighbour points. The shortest distance will be the nearest point.

The randomness of the empirical distribution is determined using, e.g., *R statistic*. The first step is to define the ratio of the average closest distance, observed to be the mean of the closest theoretical distance. It can be calculated as follows:

(6) $$R=\frac{r_{obs}}{r_{exp}}$$

Knowing *Rscale*, we can determine if the point pattern is more or less clustered than a random pattern. Of course, we have to determine the degree of this discrepancy, but generally we can claim that the smaller the *R* values (*r_obs_ < r_exp_*), the more clustered the patterns. The *Rscale* ranges from completely clustered (*R* = 0) to random (*R* = 1) and, finally, to dispersed (*R* = 2.49) [51].

For the current analysis, *r_obs_* can be associated with (*PC_Res_*) for measured data with *SP_n_* empirical value, and *r_exp_* is calculated for the theoretical, expected surface of the square.

Using *R statistic*, we should know the extent to which the observed average distance differs from the expected average distance by comparing it with the standard error *SE_r_*. Following this, we can define a calculated difference to be statistically significant when:

Probability (<95%) = (−1.96 *SE_r_*, + 1.96 *SE_r_*) (7)

To calculate *SE_r_*, we use:

(8) $$SE_{r}=\frac{0.26136}{\sqrt{N^{2}/TSP_{n}}}$$

Knowing this, we can see how the difference is compared with it by calculating a standardized *Z* score:

(9) $$Z_{R}=\frac{r_{obs}-r_{exp}}{SE_{r}}$$

If *Z_R_* > 1.96 or *Z_R_* < −1.96, we can say that the calculated difference between the observed pattern and the random pattern is statistically significant. Otherwise, although it may look somewhat clustered or dispersed visually, the pattern is not significantly different from a random pattern. Additionally, the *Z_R_* sign indicates the tendency of the pattern to be clustered or dispersed.

For further analysis, *Rscale* classification is used to determine if points are close to the regular distribution (*P_R_*) or have a tendency to be clustered or dispersed (*P_NR_*).

Knowing the point distribution, we can decide which variant would be useful for a particular target scan.

### 3.4. STEP III—Checking the Conditions and Parameters

Knowing all point cloud parameters for each checkerboard field separately, we can choose the proper variant of target centre calculation. We assume that there must be more than two points in each field. If at least one of the fields fulfils the conditions shown below, a particular variant is chosen to calculate the target centre:

(1) Variant I:

$$SRC_{1}\cup SRC_{2}\cap P_{R}\;\;\;(\mathrm{COND}.1)$$

$$SRC_{1}\cap P_{NR}\;\;(\mathrm{COND}.2)$$

In this simple case, the surface area ratio is at least 95%, and points are close to the regular distribution.

(2) Variant II:

$$SRC_{2}\cap \;P_{NR}\;\;(\mathrm{COND}.3)$$

$$SRC_{3}\cap P_{R}\cup P_{NR}\;\;(\mathrm{COND}.4)$$

This variant is adequate for fields with a lower surface area ratio <68 – 95%) and points with a tendency to be clustered or have a dispersed distribution (*RScale* ≠ 1), or generally, for fields with a very low surface ratio (68–33%).

3. Variant III:

$$SRC_{4}\cap P_{R}\cup P_{NR}\;\;(\mathrm{COND}.5)$$

This variant is suitable for cases when at least one of the checkerboard fields has a very low *SR* (below 33%).

Reassuming the above conditions, we can write them into a table (Table 2):

As shown in the results, the choice of the variant depends on the quality of the point clouds.

### 3.5. STEP IV—Solution Variants

According to the scheme in Figure 6, there are several options for point cloud processing. All of them are first based on the Surface Area Ratio and the calculation of other parameters, and then on the weighting of particular point sets. In this way, the variants are also closely related to the quality (completeness, resolution, and number of points) of point clouds, representing each square of the target.

*Variant I—regular, high-resolution point cloud*

The first solution (Figure 6) is the best possible variant, as the Surface Area Ratio (*SR*) must be higher than 95%. If both compatibility tests reach high values, the target centre can be determined by means of simply calculating the mass centre of all the points of the target (*p_i_*):

(10) $$S^{-}{}_{x}=\frac{1}{N_{x}}\sum_{i=1}^{N_{x}}p_{i}\;\;\;\;\;\;\;\;S^{-}{}_{y}=\frac{1}{N_{y}}\sum_{i=1}^{N_{y}}p_{i}\;\;\;\;\;\;S^{-}{}_{z}=\frac{1}{N_{z}}\sum_{i=1}^{N_{z}}p_{i}$$

where $S^{-}{}_{x}$—mean for *X coordinate*, $S^{-}{}_{y}$—*mean for Y coor*dinate, and $S^{-}{}_{z}$—mean for *Z* coordinate.

Otherwise, the point should be processed with one of the variants proposed below.

*Variant II—regular or irregular scan points of a lower quality*

In this variant, it is assumed that the target is scanned “in its entirety,” but with a low density or unfavourable distribution of points (Figure 7b,c). This introduces an error or makes it impossible to select the centre. It is then necessary either to reconstruct the target or to determine the point that will represent the target centre in the most precise manner. Nevertheless, the surface area ratio should not be lower than 33% (*SRC*_2_ or *SRC*_3_).

In discussing this solution, the following parameters will be taken into consideration: *PC_N_*, *PC_D_* and point cloud surface area ratio (*SR*), in order to determine the influence of particular field (square) points for target reconstruction.

First, the centres of point clouds (Formula (8)) from each field are calculated ($S_{P1}^{-},\;S_{P2}^{-},\;S_{P3}^{-},\;S_{P4}^{-})$. Subsequently, the number of points (*PC_N_*) and cloud density (*PC_D_*) are determined for each “square”. Based on those values, weights assigned to each mean of the fields are calculated, according to the following procedure.

*Weighting the Parameters*

In this solution, nonparametric statistic ranking method is used. For each of the four fields, the degree of fulfilment of the maximum for the given parameter condition (*PC_N_*, *PC_D_*, *SR*) is determined. Every condition may be fulfilled, in a range from 1 to 4, with a unit step determined as the Compliance Level (*CL*). Then, subsequent levels of fulfilment of the given condition for each square (*P_n_*) are added together, resulting in *CLP_n_*. Next, based on the obtained values, the impact of every field on the total projection of the target is determined. This will constitute the weight, which will be assigned to each mass centre of the individual field at the time of the final determination of the target centre.

Weights of the individual squares (*WP_n_*) are calculated by normalising the obtained *CL* sums. Earlier, the normalising coefficient (*NC*) is calculated on the basis of the following formula:

$$\overset{m=n}{\underset{m=1}{\sum }}\left(CLP_{n}\cdot NC\right)=1$$

(11) $$NC=\frac{1}{\sum_{1}^{n}CLP_{n}}$$

(12) $$WP_{n}=CLP_{n}\cdot NC$$

Therefore, the new values of the target centre coordinates (X’, Y’, Z’) are calculated on the basis of the weighted mass centres of all the fields:

(13) $$\begin{array}{c} X^{\prime }=\frac{X_{S_{P1}^{-}}\cdot WP_{1}+X_{S_{P2}^{-}}\cdot WP_{2}+X_{S_{P3}^{-}}\cdot WP_{3}+X_{S_{P4}^{-}}\cdot WP_{4}}{WP_{1}+WP_{2}+WP_{3}+WP_{4}}\\ Y^{\prime }=\frac{Y_{S_{P1}^{-}}\cdot WP_{1}+Y_{S_{P2}^{-}}\cdot WP_{2}+Y_{S_{P3}^{-}}\cdot WP_{3}+Y_{S_{P4}^{-}}\cdot WP_{4}}{WP_{1}+WP_{2}+WP_{3}+WP_{4}}\\ Z^{\prime }=\frac{Z_{S_{P1}^{-}}\cdot WP_{1}+Z_{S_{P2}^{-}}\cdot WP_{2}+Z_{S_{P3}^{-}}\cdot WP_{3}+Z_{S_{P4}^{-}}\cdot WP_{4}}{WP_{1}+WP_{2}+WP_{3}+WP_{4}} \end{array}$$

An example of this step is shown in Table 3.

The fields that, in the “impact ranking,” have the smallest values—have also the smallest weights assigned to the resultant calculations.

*Variant III—irregular scan points, low completeness level*

**Figure 9:**
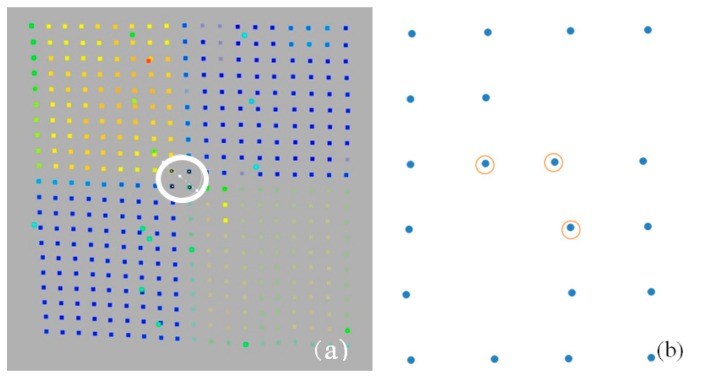
Examples of the “inner extreme points” of the target point cloud (**a**) regular points; (**b**) irregular and missing points.

Targets are scanned with a low resolution or are incomplete (missing centre points or most edge points, SRC_4_). In this approach, the edge points of the set of points of individual polygons and the “inner extreme points” are first determined (circled in white in Figure 9a and in orange in Figure 9b). These points belong to the corners of each of the checkerboard fields (squares/polygons) and are the closest points to the target centre.

**Figure 10:**
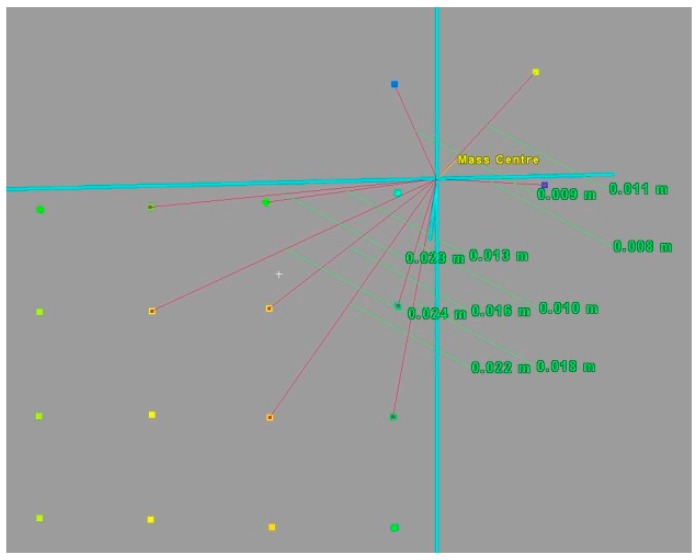
Example of the nearest neighbour analysis for inner extreme points.

These points are selected from the set of boundary points by determining the minimum and maximum of the set (1). Next, the mass centre ($S^{-}$) of all the points representing the entire target (all fields) is determined. Then, the Euclidean distances from $S^{-}$ to the neighbouring points *p_n_* (Figure 10) are calculated $d\left|S^{-},p_{n}\right|$.

Points are accepted as internal extreme points (IEP) if:

$$d\left|S^{-},p_{n}\right|\;\leq \;1.96\;\mathrm{SD}\;\;\;(\mathrm{COND}.6)$$

If the number of points with similar or identical distance and scan resolution is lower than 4, the inner extreme points cannot serve as the basis for directly identifying the target centre (Figure 10). They will then be subject to weighting on the basis of the surface areas of individual squares (*SP*_1_). The centre points are assigned the weights, calculated as in variant II, with the difference that the “inner extreme points,” instead of mass centres, are weighted in this variant.

## 4. Results and Discussion

As depicted in the introduction of this paper, point cloud-based solutions were considered in the development of the approach. The developed approach is applied to a few datasets within the same scan project in order to draw conclusions regarding the degree of automation and the transferability of the approach.

The subject of the assessment was the geometric quality of the target centre identification using different methods. The assessment of the proposed approach is divided into two parts: assessment relative to the reference (terrain) measurements and assessment relative to different methods—manual point identification, mean of all points, and the automatic detection of the target centre.

In the experiment, over 20 different types of targets, acquired from different scan stations of a lower quality, were used.

A proposed workflow (Figure 6) has been calculated for all of the chosen points. Part of the results are described briefly below. First, all cloud parameters for each target field were defined. Then, Surface Area Ratio and *Rscale* classification were conducted. In Table 4, the results for the chosen targets are presented.

For targets marked in grey, the *Rscale* is close to 2.15, and the validation of the statistic, the *Z* score, indicates a regular point pattern.

**Figure 11:**
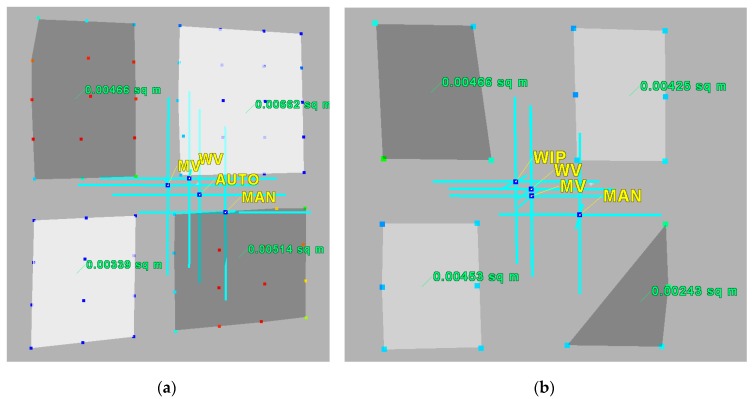
The centres identified with different solutions: *MV*—mass centre of all target points, *WV*—target centre determined by the variant II approach, *WIP*—variant III, *MAN*—manual identification of the target centre, and *AUTO*—automatic. (**a**) Target 97 (regular, good quality), (**b**) target 106 (incomplete, irregular).

Next, for all targets, new centres are calculated. In Figure 11, an example of the workflow results for two of the targets (97 and 106) is presented. The abbreviations indicate the centres identified with different solutions: *MV*—mass centre of all target points, *WV*—target centre determined by variant II, *WIP*—by variant III, *MAN*—manual identification of the target centre, and *AUTO*—automatic detection of the centre. Polygons represent the detected *B*&*W* checkerboard fields.

According to the conditions (COND.1-5), it can be seen that, for target 97, COND.1 indicates that variant I of the target centre solution should be used. Similarly, for target 106, COND.5 indicates that variant III of the target centre solution should be used. The numerical results are presented in the table below.

**Figure 12:**
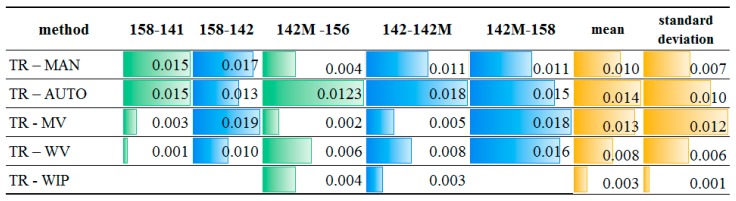
Accuracy check for the chosen targets.

**Figure 13:**
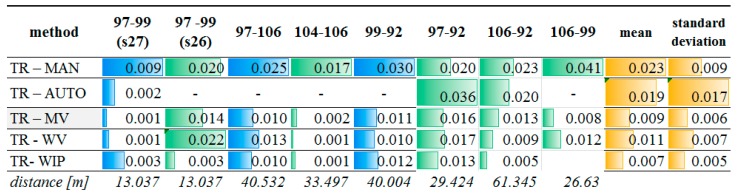
Accuracy check for the chosen low-quality targets.

Due to the fact that the scanning data are measured in a different coordinate system from the reference data, the values of the distance between the target centres calculated on the basis of the designated coordinates were used for the purposes of control and comparative analysis. The values for the selected distances are presented in Figure 12 and Figure 13. Differences in the distance lengths are presented as absolute values.

When comparing the values listed in Figure 12, it can be seen that the values, obtained as a result of calculating the accuracy of the point clouds using the proposed methods, are close to those obtained by means of the total station measurements. The accuracy of the terrain measurements reaches a standard deviation of 3–4 mm. The smallest differences are in the distances calculated on the basis of coordinates, determined with two variants of the weighted mean method (*WV* and *WIP*). In the case of targets scanned with a good quality (141, 142, and 156), the smallest discrepancies are observable for the *WV* method, ranging from 1 to 18 mm, and for targets with an assumed lower accuracy (158), the accuracies are even higher (in the range of 1 and 10 mm). The results are comparable to the accuracies obtained by the manual measurement (*MAN*) and ranges from 0 to 17 mm. The means and standard deviations for both methods do not exceed 10 mm.

It can therefore be concluded that the results of the proposed methods for the automatic identification of the target centre are close in accuracy to those of the manual measurement. At the same time, in comparison with other automatic methods of checkerboard target centre detection, they are approximate 40% more accurate.

Similar comparisons have been conducted for targets with assumed low-quality scans. Different configurations of distances between these points and points of better quality were calculated. The results are shown in Figure 13.

Again, the results of the proposed methods for the improvement of the automatic identification of the target centre are close in accuracy to those of the manual measurement and particularly in those cases when automatic detection is impossible. The standard deviation ranges from 9 to 17 mm for the proposed methods, while using other methods, it ranges from 5 to 7 mm.

The adequate choice of the variant determines the quality of the results. This is particularly visible in the case of points 97 and 99. According to COND.1-5, targets 97 and 99 (s26) should be calculated using variant III. Indeed, the discrepancy in the distance between these two points is critically lowest.

**Figure 14:**
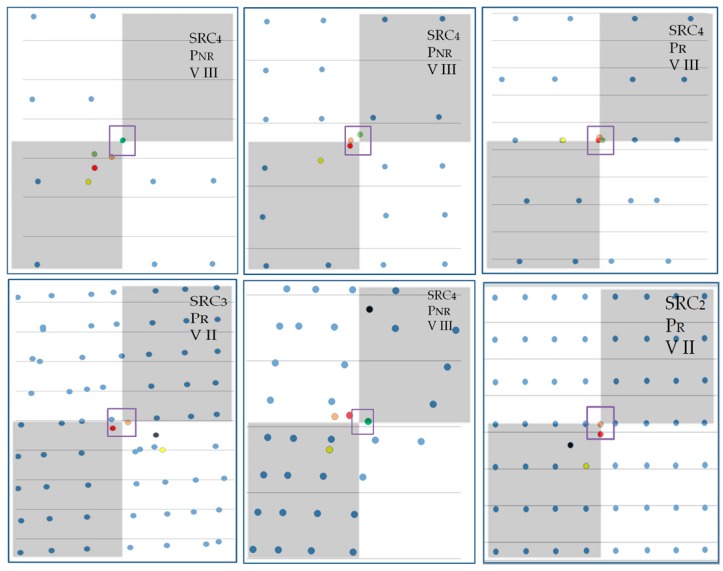
Final results for the chosen targets in comparison to the real target image. Upper—low-quality scans, lower—regular and irregular point distribution. Black dots—automatic identification, yellow—manual, red—variant I, orange—variant II, green—variant III.

Some other examples of final results using different variants in different targets are shown below (Figure 14). Methods of target centre determination are color-coded: black—automatic, yellow—manual, red—mean value/mass (variant I), orange—weighted mean value (variant II), green—weighted inter extreme points (*WIP*).

In the upper-right corners of each target, the Surface Area Ratio class, *Rscale* class and preferred variant are given. In almost all cases, the proposed original methods gave better results in target centre identification than the automatic or manual methods.

## 5. Conclusions

The main goal of this work was to propose an easy and alternative method to improve target centre identification using weighted point cloud parameters. This could be useful in cases where there is no possibility of secondary measurement, when the number of targets is too low or the scans are of a low accuracy (distance too long or unfavourable scan angle). It could be helpful to improve the quality of target identification. Precise, fully automatic identification is not possible, but it is important to be aware of its quality, particularly in the context of further processing (georeferencing or registration). What is more, preliminary, initial semi-automatic centre detection can be improved using the proposed solutions.

The metrics for the assessment of the results were also proposed. First, modified Point Pattern Analysis and Surface Ratio classification were presented. The standard deviations related to the distance between test points and reference check points were proposed as a quality index. Therefore, weight-based approaches are proposed. These tools are essential for quality assessment and detection improvement, since a complete automation of target centre detection seems to be insufficient for poorly scanned targets. Thus, the developed approach responds positively to the three items considered, namely, the transferability, the degree of automation and the geometric quality. The results of the proposed methods for the automatic identification of the target centre are close in accuracy to those of the manual measurement. At the same time, the proposed methods checkerboard target centre detection from low-quality point clouds are approximate 40% more accurate than automatic and manual methods. Further research will focus on improvement of proposed solutions mainly in automatic preliminary target shape sub-selection.

## Figures and Tables

**Table 1:** Chosen parameters of the target point clouds (142, 156—good quality, 106—low quality).

	142				156				106			
	*SR P*_1_	*SR P*_2_	*SR P*_3_	*SR P*_4_	*SR P*_1_	*SR P*_2_	*SR P*_3_	*SR P*_4_	*SI P*_1_	*SI P*_2_	*SI P*_3_	*SI P*_4_
*SR* (%)	78	75	81	88	93	82	87	102	58	52	30	56
*PC_N_*	101	94	99	100	35	33	33	37	4	6	4	6
*PC_D_* (p/cm^2^)	1.6	1.6	1.5	1.4	0.5	0.5	0.5	0.4	0.09	0.14	0.16	0.13
*PC*_Res_ (cm)	0.8	0.9	0.8	0.8	1.3	1.5	1.5	1.6	4.6	3.8	4.3	3.9

**Table 2:** Choice of the variant according to the surface and regularity class.

	*SRC*_1_	*SRC*_2_	*SRC*_3_	*SRC*_4_
*P_R_*	*I*	*I*	*II*	*III*
*P_NR_*	*I*	*II*	*II*	*III*

**Table 3:** List of parameters for individual fields for regular, good quality target scans (142) and for irregular, incomplete target scans (97).

**Target 142 (Good Quality)** ***B* and *W* Polygons Parameters**			**Target 142 (Good Quality)** ***B* and *W* Polygons *CL* and Weights**			
**Surface Area**	**Number of Points**	**Density**	***P*_1_**	***P*_2_**	***P*_3_**	***P*_4_**
*P*_1_	*P*_1_	*P*_2_	4	4	3	1
*P*_3_	*P*_2_	*P*_3_	4	3	3	1
*P*_2_	*P*_3_	*P*_1_	2	2	2	1
*P*_4_	*P*_4_	*P*_4_	*WP*1 = 0.33	*WP*2 = 0.30	*WP*3 = 0.27	*WP*4 = 0.10
**Target 97 (Low Quality)** ***B* and *W* Polygons Parameters**			**Target 97 (Low Quality)** ***B* and *W* Polygons *CL* and Weights**			
**Surface Area**	**Number of Points**	**Density**	***P*_1_**	***P*_2_**	***P*_3_**	***P*_4_**
*P*_4_	*P*_4_	*P*_4_	3	3	1	4
*P*_1_	*P*_1_	*P*_2_	3	2	1	4
*P*_2_	*P*_2_	*P*_1_	2	2	1	4
*P*_3_	*P*_3_	*P*_3_	*WP*1 = 0.27	*WP*2 = 0.23	*WP*3 = 0.10	*WP*4 = 0.40

**Table 4:** Results of surface and regularity tests for the chosen targets.

	*97 s 27*	*97 s 26*	*99 s 27*	*99 s 26*	*106 s 28*	*104 s 28*	*106 s 27*	*92 s 26*
*r obs*	0.014	0.037	0.024	0.043	0.061	0.015	0.041	0.022
*r exp*	0.009	0.017	0.013	0.020	0.028	0.010	0.021	0.010
*Rscale*	1.60	2.15	1.82	2.15	2.15	1.49	2.00	2.160
*PC_N_*	105	27	63	20	10	219	20	55
*Z score*	20.030	3.976	−0.806	0.745	5.963	−4.773	8.520	1.391
*SR class*	2	4	3	4	4	2	4	3
*Rscale class*	P_NR_	P_NR_	P_R_	P_R_	P_NR_	P_NR_	P_NR_	P_R_
